# A forkhead transcription factor contributes to the regulatory differences of pathogenicity in closely related fungal pathogens

**DOI:** 10.1002/mlf2.12011

**Published:** 2022-03-24

**Authors:** Weixin Ke, Yuyan Xie, Yue Hu, Hao Ding, Xin Fan, Jingjing Huang, Xiuyun Tian, Baokun Zhang, Yingchun Xu, Xiao Liu, Ying Yang, Linqi Wang

**Affiliations:** ^1^ State Key Laboratory of Mycology, Institute of Microbiology Chinese Academy of Sciences Beijing China; ^2^ College of Life Sciences University of Chinese Academy of Sciences Beijing China; ^3^ Department of Infectious Diseases and Clinical Microbiology, Beijing Chaoyang Hospital Capital Medical University Beijing China; ^4^ Department of Clinical Laboratory, Peking Union Medical College Hospital, Peking Union Medical College Chinese Academy of Medical Sciences Beijing China; ^5^ Beijing Key Laboratory for Mechanisms Research and Precision Diagnosis of Invasive Fungal Diseases, Peking Union Medical College Hospital, Peking Union Medical College Chinese Academy of Medical Sciences Beijing China; ^6^ Graduate School, Peking Union Medical College Chinese Academy of Medical Sciences Beijing China; ^7^ Beijing Key Laboratory of New Molecular Diagnosis Technologies for Infectious Disease, Department of Biotechnology Beijing Institute of Radiation Medicine Beijing China

**Keywords:** *Cryptococcus deuterogattii*, *Cryptococcus neoformans*, forkhead transcription factor, pathogenicity

## Abstract

*Cryptococcus neoformans* and its sister species *Cryptococcus deuterogattii* are important human fungal pathogens. Despite their phylogenetically close relationship, these two *Cryptococcus* pathogens are greatly different in their clinical characteristics. However, the determinants underlying the regulatory differences of their pathogenicity remain largely unknown. Here, we show that the forkhead transcription factor Hcm1 promotes infection in *C. neoformans* but not in *C. deuterogattii*. Monitoring *in vitro* and *in vivo* fitness outcomes of multiple clinical isolates from the two pathogens indicates that Hcm1 mediates pathogenicity in *C. neoformans* through its key involvement in oxidative stress defense. By comparison, Hcm1 is not critical for antioxidation in *C. deuterogattii*. Furthermore, we identified *SRX1*, which encodes the antioxidant sulfiredoxin, as a conserved target of Hcm1 in two *Cryptococcus* pathogens. Like *HCM1*, *SRX1* had a greater role in antioxidation in *C. neoformans* than in *C. deuterogattii*. Significantly, overexpression of *SRX1* can largely rescue the defective pathogenicity caused by the absence of Hcm1 in *C. neoformans*. Conversely, Srx1 is dispensable for virulence in *C. deuterogattii*. Overall, our findings demonstrate that the difference in the contribution of the antioxidant sulfiredoxin to oxidative stress defense underlies the Hcm1‐mediated regulatory differences of pathogenicity in two closely related pathogens.

## INTRODUCTION

*Cryptococcus neoformans* and *Cryptococcus deuterogattii* (formerly *C. gattii*, genotype AFLP6/VGII) represent the most important human fungal pathogens in the phylum Basidiomycota, which contains more than 30,000 known species^1^, ^2^. These pathogenic species are evolutionally close and have nearly identical asexual and sexual life cycles^3^, ^4^. Asexually, *C. neoformans* and *C. deuterogattii* mostly proliferate as yeast cells and reproduce by budding. Under favorable conditions, fungi of both species undergo similar sexual differentiation to produce infectious basidiospores, which can be inhaled into the lungs^4^. Both *C*. *neoformans* and *C*. *deuterogattii* have exceptional capabilities for lung colonization and dissemination from the lung to the brain, causing pneumonia and meningitis^5^, ^6^, ^7^. In addition to their importance as deadly pathogens, with advances in genetics, large‐scaled deletion mutant libraries, whole‐genome sequencing, and murine models^8^, ^9^, ^10^, ^11^, ^12^, ^13^, ^14^, these two closely related fungi have become excellent models for investigation of the genetic and regulatory bases of fungal pathogenesis, biology, and evolution.

Previous studies have identified multiple common virulence traits in *C. neoformans* and *C. deuterogattii*. For instance, both of them can multiply well at typical mammalian body temperatures, which is believed to distinguish them from nonpathogenic *Cryptococcus* species^15^, ^16^. In addition, they both are able to produce capsules and melanin, which serve as the major virulence factors that facilitate their infectivity^17^, ^18^. Despite these shared virulence traits, *C. neoformans* and *C. deuterogattii* are greatly different in their genetic, ecological, clinical, and stress adaptation characteristics^19^, ^20^, ^21^. Different from the sister species *C. deuterogattii*, which is often associated with plants, *C. neoformans* is frequently detected in pigeon droppings^14^, ^22^. Furthermore, *C. neoformans* is one of the most common fungal pathogens causing infections in immunocompromised individuals, whereas *C. deuterogattii* is the causative agent for an ongoing cryptococcosis outbreak in the Pacific Northwest regions that commonly infects immunocompetent patients^23^, ^24^, ^25^. These differences likely mirror their distinct adaptation abilities to diverse stress from natural niches or hosts. Consistent with this hypothesis, an earlier study indicated a better proliferation of R265, the model strain of *C. deuterogattii*, in macrophages compared to the *C. neoformans* reference strain H99. The better proliferation of R265 was demonstrated to be attributed to its more effective defense against reactive oxygen species (ROS) and nitrosative stress within macrophages^26^, ^27^, ^28^. These dissimilarities strongly suggest that there exist regulatory differences of pathogenicity in these phylogenetically close pathogens. However, the determinants involved remain largely unknown.

In this study, we show that the forkhead regulator Hcm1 plays an important regulatory role in the pathogenicity of *C. neoformans* but not in that of *C. deuterogattii*. This difference is largely due to the different contributions of Hcm1 to antioxidative defense in two *Cryptococcus* species. We found that *C. neoformans*‐derived Hcm1, but not its *C. deuterogattii* homolog, plays a critical role in oxidative stress responses. Chromatin immunoprecipitation (ChIP)–quantitative polymerase chain reaction (qPCR) and transcriptional assays together with epistasis assessment identified the antioxidant sulfiredoxin gene *SRX1* as the conserved target of Hcm1. Likewise, the loss of *SRX1* showed a more severe defect in oxidative stress defense in *C. neoformans* than in *C. deuterogattii*. Importantly, the overexpression of *SRX1* can largely restore the pathogenicity of *C. neoformans* strain devoid of Hcm1. In contrast, Srx1 is dispensable for virulence in *C. deuterogattii*. Overall, our data demonstrate that Hcm1 is a critical determinant, which contributes to the regulatory differences of pathogenicity in *C. neoformans* and *C. deuterogattii*.

## RESULTS

### Hcm1 is not involved in cell cycle regulation in pathogenic *Cryptococcus* species

**Figure 1:**
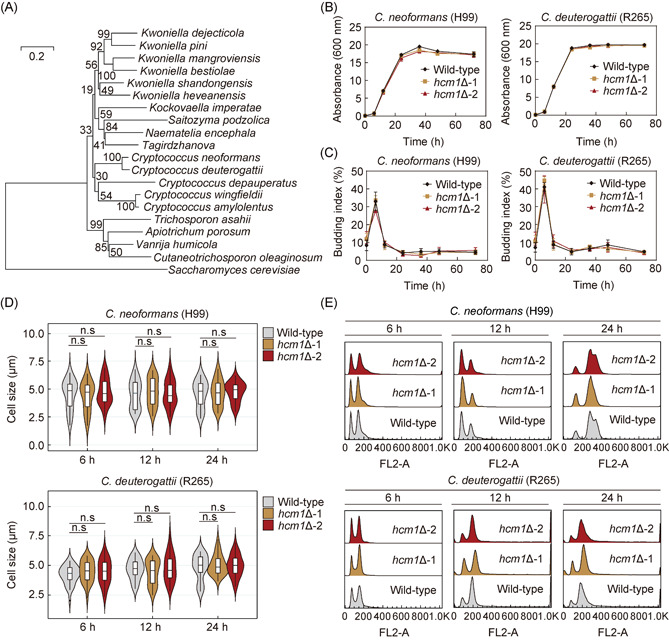
Hcm1 is not involved in cell cycle regulation in *Cryptococcus* pathogens. (A) Phylogenetic analysis of Hcm1 homologs. The phylogenetic tree was generated with MEGA‐X using the neighbor‐joining method. The bar marker indicates the genetic distance proportional to the number of amino acid substitutions. Bootstrap values based on 1000 replications are indicated at the branch points. Growth curves (B) and budding index (C) of the wild‐type and *hcm1*Δ mutants in the *Cryptococcus neoformans* H99 and *Cryptococcus deuterogattii* R265 background. (D) Violin plots showing the cell size distribution of wild‐type and *hcm1*Δ mutant strains. *Cryptococcus* cells were grown in YPD liquid medium for 6, 12, and 24 h (*n* > 50). (E) Fluorescence‐activated cell sorting (FACS) analysis of propidium iodide‐stained wild‐type and *hcm1*Δ mutant cells cultured in YPD liquid medium for 6, 12, and 24 h. n.s, not significant. YPD, yeast extract–peptone–dextrose.

CNAG_03116 is a forkhead transcription factor that is highly conserved in species belonging to Cryptococcaceae (Figure 1A), and this protein is named Hcm1 because it shares a modest similarity with Hcm1 from *Saccharomyces cerevisiae*, where it is a key regulator of cell cycle progression^29^, ^30^, ^31^, ^32^, ^33^. However, the conserved sequence shared between CNAG_03116 and *S. cerevisiae* Hcm1 is only within the forkhead domain and CNAG_03116 does not contain phosphorylation sites outside the forkhead region, which is critical for the activity of *S*. *cerevisiae* Hcm1 in cell cycle control^30^, ^34^. These data raise the possibility that CNAG_03116 may not be important for cell cycle regulation in *C. neoformans*. To test this idea, we deleted its coding gene in the *C. neoformans* clinical reference strain (H99). In *S. cerevisiae*, the *hcm1*Δ mutant exhibited a delayed G2 phase^33^, which resulted in growth retardation. However, we found that deletion of the CNAG_03116 coding gene did not affect the growth or proliferation of *C. neoformans* (Figure 1B,C), consistent with the findings reported by Jung et al^11^. Additionally, phenotypic assessment combined with flow cytometry analysis indicated that the absence of CNAG_03116 in *C. neoformans* neither led to G2 phase delay nor caused cell size alterations (Figure 1D,E). These results demonstrate the independence of CNAG_03116 in cell cycle regulation in *C. neoformans*, at least under the conditions we tested. Furthermore, we further knocked out the gene encoding the homolog of CNAG_03116 in the *C. deuterogattii* clinical reference strain (R265) using the transient CRISPR‐Cas9 coupled with electroporation (TRACE) system^35^. Gene knockout did not alter cell growth, cell size, and cell cycle progression (Figure 1B–E). These results illustrate that, unlike Hcm1 in *S. cerevisiae*, its orthologs in *Cryptococcus* pathogens are not required for cell cycle progression. Despite this, to avoid unnecessary confusion, we still used the original name of the gene (*HCM1*) in this study.

### Hcm1 mediates the regulatory differences of pathogenicity in different *Cryptococcus* species

**Figure 2:**
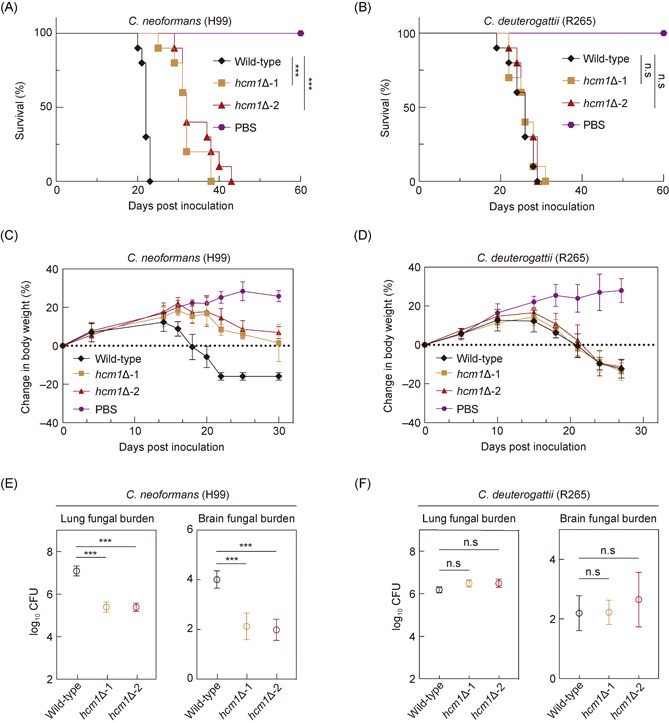
Hcm1 promotes pathogenicity in *Cryptococcus neoformans* but not in *Cryptococcus deuterogattii*. Ten C57 BL/6 female mice were infected with wild‐type and *hcm1*Δ mutant strains in *C. neoformans* H99 (A) and *C. deuterogattii* R265 (B) backgrounds. The survival percentage of infected mice and uninfected mice was monitored over 60 days postinfection. Dynamic curves of the body weight changes in mice infected with wild‐type and *hcm1*Δ mutant strains in *C. neoformans* H99 (C) and *C. deuterogattii* R265 (D) backgrounds, respectively. C57 BL/6 female mice were infected with the wild‐type and *hcm1*Δ mutant strains in *C. neoformans* H99 (E) and *C. deuterogattii* R265 (F) backgrounds. Fungal burden in the lungs and the brains was assessed by counting CFU at DPI 14. Data represent the mean ± SD (*n* = 3). ***p < 0.001, and n.s, not significant. CFU, colony forming unit; DPI, days postinoculation; PBS, phosphate‐buffered saline; SD, standard deviation.

To investigate the impact of Hcm1 on pathogenicity in different *Cryptococcus* pathogens, we took advantage of the intranasal mouse model that mimics human cryptococcal disease^8^. Mortality curves revealed that the two independent *hcm1*Δ mutants generated from H99 displayed hypovirulent profiles (Figure 2A). In agreement, the rates of weight loss of mice infected by these mutants were significantly slower than that of mice inoculated with the parent strain (Figure 2C). Besides, we found that the fungal burdens in the lungs and brains of mice infected with the *hcm1*Δ mutant were significantly lower than those inoculated with the H99 wild‐type strain (Figure 2E). Moreover, morphological observations and pathological tissue section analyses of lungs at 14 days postinoculation (DPI) showed that mice infected with the *hcm1*Δ mutant strain caused less damage and immune cell infiltration than the mice infected with the H99 wild‐type strain (Figure S1A,B). These data indicate that Hcm1 facilitates pathogenicity in the *C. neoformans* H99. Interestingly, unlike in the clinical reference strain of *C. neoformans* H99, deletion of *HCM1* in R265 did not cause significant changes in mortality rates (Figure 2B). Consistently, similar weight loss rates and fungal burdens were observed in mice infected with the R265 wild‐type and the corresponding *hcm1*Δ mutant strains (Figure 2D,F). Thus, Hcm1 is dispensable for virulence in *C. deuterogattii* R265.

### Hcm1 contributes differentially to oxidative stress defense in different *Cryptococcus* pathogens

**Figure 3:**
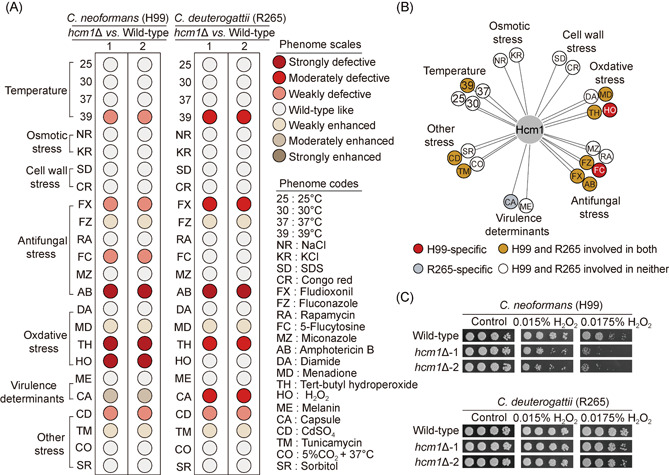
Hcm1 contributes more strongly to oxidative stress defense in *Cryptococcus neoformans* H99 than in *Cryptococcus deuterogattii* R265. (A) Semiquantitatively phenotypic assays of hcm1Δ mutants constructed in *C. neoformans* H99 and *C. deuterogattii* R265 backgrounds based on data presented in Figure S2. Red and yellow circles represent defective and enhanced, respectively. The gradients of red or yellow indicate the phenotype strengths (strong, intermediate, and weak). (B) Phenotypic comparison of the *hcm1*Δ mutants constructed in *C. neoformans* H99 and *C. deuterogattii* R265 backgrounds, based on data presented in Figure S2. (C) Cells were grown overnight in YPD at 30°C, five‐fold serially diluted, spotted onto YPD medium containing H_2_O_2_ at the indicated concentrations, and incubated for 2–3 days before photographing.

To understand why Hcm1 contributes differentially to virulence in H99 and R265, we performed comparative phenotypic analysis. To this end, H99 and R265 together with their corresponding *hcm1*Δ mutants (two independent mutants were applied for each pathogen) were examined for their phenotypic traits under 24 distinct growth conditions. The phenotypic assessment showed that the H99‐derived *hcm1*Δ mutants produced phenotypes that were highly similar to those reported by Jung et al.^11^, indicating the reliability of our phenotypic analysis (Figures 3A and S2A). We noticed that loss of Hcm1 did not impair the major *Cryptococcus* virulence factors in H99, including adaptation to host temperature (37°C) as well as production of melanin and capsule (Figure S3A–C), suggesting that these pathogenicity traits are independent of Hcm1‐mediated virulence in H99. For R265, *hcm1*Δ likewise displayed an undetectable change in phenotypic traits in growth at 37°C or melanin synthesis but had a modestly reduced capsule thickness (Figure S3A–C). Considering that Hcm1 is dispensable for pathogenicity in R265, such reduction may be insufficient to affect the outcome of infection. These data suggest that the differences in Hcm1‐mediated pathogenicity are unlikely to be due to these well‐recognized *Cryptococcus* virulence factors. On the basis of our phenotypic evaluations, *hcm1*Δ mutants from H99 or R265 exhibited similar profiles of phenotypic traits, except for capsule formation as well as resistance to hydrogen peroxide (H_2_O_2_) and 5‐flucytosine (Figure 3B). We focused on the phenotype associated with H_2_O_2_ tolerance, which has been well established to be critically linked with pathogenicity in various fungal pathogens^36^, ^37^. As shown in Figure 3C, deletion of *HCM1* in H99 drastically attenuated growth fitness upon exposure to H_2_O_2_, whereas there was nearly indistinguishable growth difference between R265 and its corresponding *hcm1*Δ mutant strains under treatment with H_2_O_2_ at the same concentration. These findings demonstrate that Hcm1 homologs from H99 or R265 are distinct in their contribution to oxidative stress defense, which is important for fungal survival within host immune cells^36^, ^38^.

### Hcm1 promotes antioxidation and virulence through its direct control of sulfiredoxin Srx1 in *C. neoformans*

**Figure 4:**
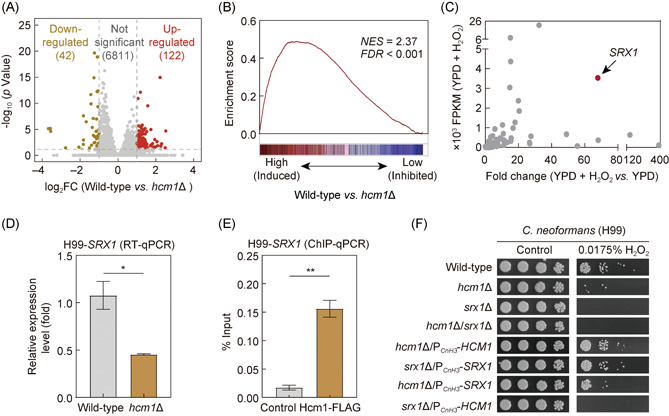
Hcm1 promotes antioxidation through its direct control of the sulfiredoxin Srx1 in *Cryptococcus neoformans*. (A) Volcano diagram of the differentially expressed genes in comparison with the wild‐type and *hcm1*Δ mutant strain in the *C. neoformans* H99 background. (B) Gene Set Enrichment Analysis demonstrated that enrichment of H_2_O_2_‐induced genes in the targets was transcriptionally activated by *C. neoformans* Hcm1. (C) Transcriptional induction response of Hcm1 targets to hydrogen peroxide. The red dot indicates the sulfiredoxin‐coding gene *SRX1*. The original transcriptome data for DEGs in response to hydrogen peroxide were obtained from a previous study^39^. (D) RT‐PCR analysis of *SRX1* transcription in different strains from H99 background. Data represent the mean ± SD (*n* = 3). (E) Chromatin immunoprecipitation (ChIP) was performed with an anti‐FLAG antibody in *hcm1*Δ cells in which the FLAG‐fused Hcm1 was overexpressed. ChIP enrichment was detected by qPCR across an approximately 220 bp region upstream of the *SRX1* ORF. Data represent the mean ± SD (*n* = 3). (F) Spotting susceptibility assays of wild‐type, *hcm1*Δ, *srx1*Δ, *hcm1*Δ/*srx1*Δ, *hcm1*Δ/P_*CnH3*_‐*HCM1*, *srx1*Δ/P_*CnH3*_‐*SRX1*, *hcm1*Δ/P_*CnH3*_‐*SRX1*, and *srx1*Δ/P_*CnH3*_‐*HCM1* strains in the *C. neoformans* H99 background were performed in the presence of H_2_O_2_ at the indicated concentration. *p < 0.05, **p < 0.01. DEGs, differentially expressed genes; FC, 5‐flucytosine; FDR, false discovery rate; NES, normalized enrichment score; ORF, open reading frame; qPCR, quantitative polymerase chain reaction; RT‐PCR, reverse transcription polymerase chain reaction.

To reveal the mechanism underlying Hcm1‐mediated defense against oxidative stress in *C. neoformans*, we performed a comparative transcriptome analysis via high‐coverage RNA sequencing (RNA‐seq), which targets the *hcm1*Δ mutant and its parent strain H99. We analyzed 6892 genes predicted to encode proteins, and thus our analysis covered 98.8% of the protein‐coding genes in the entire genome. Among these genes, 164 differentially expressed genes (DEGs) were identified in response to Hcm1 deficiency, including 42 and 122 genes, downregulated or upregulated by Hcm1 (|log_2_(fold change)| > 1.0, *q* < 0.05) (Figure 4A). Notably, we did not detect enrichment of cell cycle‐related genes in Hcm1 regulon, again supporting the conclusion that Hcm1 is not important for cell cycle progression in *C*. *neoformans*.

To evaluate the importance of the targets of Hcm1 in oxidative stress defense, we reanalyzed the publicly available transcriptomic data generated by Cheon et al., which aimed to identify DEGs of *C*. *neoformans* in response to H_2_O_2_ ^39^. It was shown that the genes induced in response to H_2_O_2_ are significantly overrepresented in the Hcm1‐induced targets (Figure 4B). These results again support the importance of Hcm1 in the oxidative stress response in *C. neoformans*. We noticed that among the targets of Hcm1, the sulfiredoxin‐coding gene *SRX1* displayed highly induced and abundant transcription in the presence of H_2_O_2_ (Figure 4C). In fungi, Srx1 homologs generally play important roles in the recycling of hyperoxidized form (R‐SO_2_H) of peroxiredoxins, which reduce inorganic and organic peroxides using electrons donated by reduced thioredoxin^40^, ^41^, ^42^. Upadhya et al. have demonstrated that in H99, the *srx1*Δ mutant was hypersensitive to H_2_O_2_. In addition, their animal experiments demonstrated that the knockout of *SRX*1 in H99 significantly weakened pathogenicity^43^. These phenotypes were highly similar to those observed in the *hcm1*Δ mutant strains. These findings led us to ask whether *SRX1* acts as a key target of Hcm1 in oxidative stress defense and virulence in *C. neoformans*.

**Figure 5:**
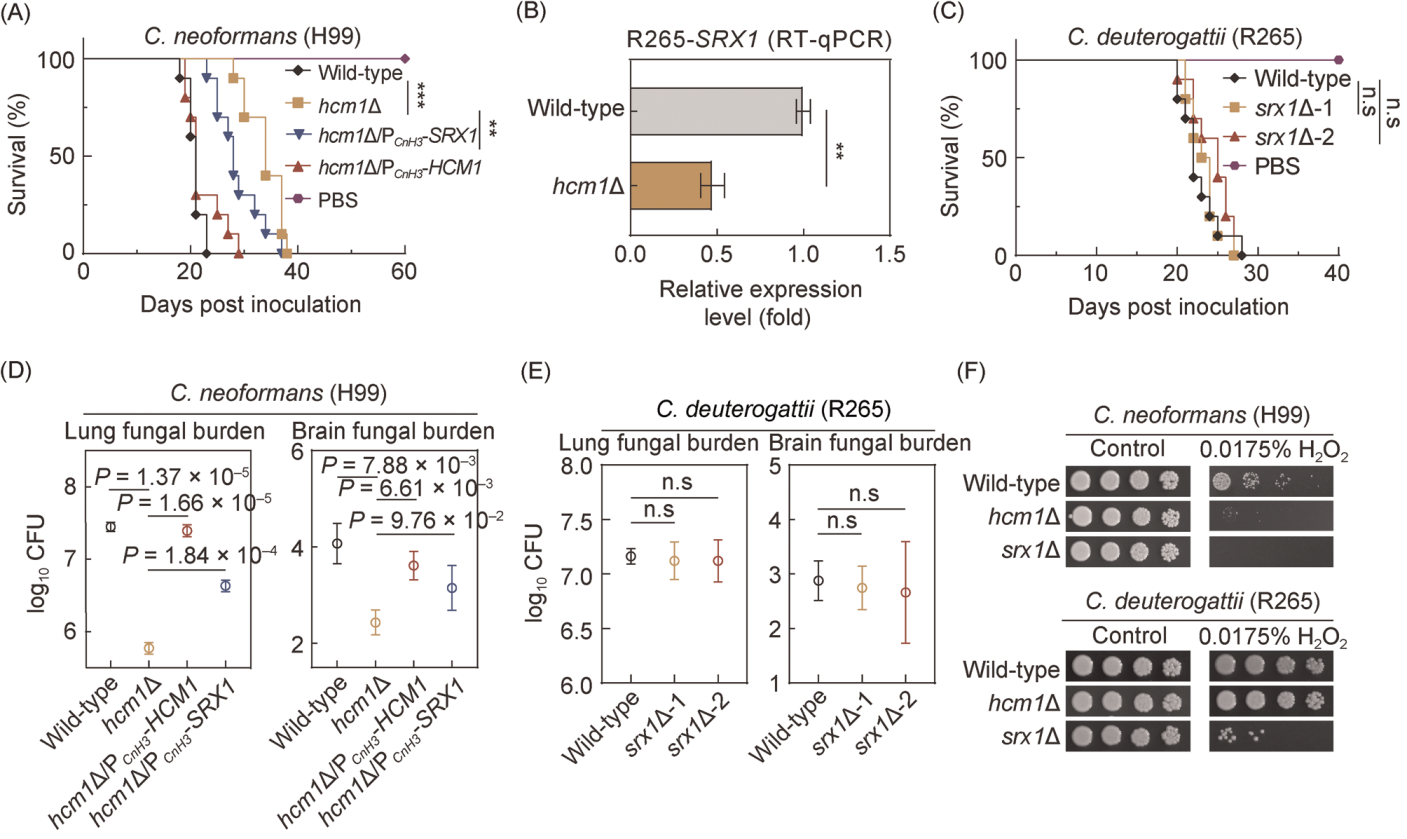
*SRX1* as a downstream target of Hcm1 promotes virulence in H99 but not in R265. (A) Survival curves plotted against time upon challenge with wild‐type, *hcm1*Δ, *hcm1*Δ/P_*CnH3*_‐*HCM1*, and *hcm1*Δ/P_*CnH3*_‐*SRX1* strains in the *Cryptococcus neoformans* H99 background by intranasal instillation. (B) RT‐PCR analysis of *SRX1* transcription in wild‐type and *hcm1*Δ strains in the *Cryptococcus deuterogattii* R265 background. (C) Survival curves plotted against time upon challenge with wild‐type and *srx1*Δ strains in the *C. deuterogattii* R265 background by intranasal instillation. (D) Lung and brain fungal burdens from mice that were infected with wild‐type, *hcm1*Δ, *hcm1*Δ/P*_CnH3_*‐*HCM1* and *hcm1*Δ/P_*CnH3*_‐*SRX1* in the *C. neoformans* H99 background at DPI 14. (E) Lung and brain fungal burdens from the mice that were infected with wild‐type and *srx1*Δ strains in the *C. deuterogattii* R265 background at DPI 14. (F) Spotting susceptibility assays of wild‐type, *hcm1*Δ, and *srx1*Δ strains in H99 and R265 backgrounds were performed in the presence of H_2_O_2_ at the indicated concentration. Data represent the mean ± SD (*n* = 3). **p < 0.01, ***p < 0.001, and n.s, not significant.

To test this idea, we first performed reverse transcription polymerase chain reaction (RT‐PCR)‐based transcriptional analyses, which confirmed the transcriptomic result, indicating the importance of Hcm1 in activating the expression of *SRX1* in H99 (Figure 4D). A ChIP‐qPCR analysis using an anti‐FLAG antibody further revealed a significant enrichment of FLAG‐labeled Hcm1 at the promoter of *SRX1* (Figure 4E), suggesting that Hcm1 controls the expression of *SRX1* in a direct manner. Moreover, we performed epistasis analysis for determining the genetic relationship between *HCM1* and *SRX1* in terms of antioxidation and virulence in *C. neoformans*. To achieve this, we constructed an H99‐derived *hcm1*Δ/*srx1*Δ double mutant strain and overexpressed *SRX1* and *HCM1* in the *hcm1*Δ mutant and *srx1*Δ mutant, respectively. It was shown that the *hcm1*Δ/*srx1*Δ double mutant displayed a defective growth fitness in the presence of H_2_O_2_ at a level similar to *srx1*Δ, which was more sensitive to oxidative stress than *hcm1*Δ. In addition, overexpression of *SRX1* restored the defect of *hcm1*Δ in defense against oxidative stress, but not *vice versa* (Figure 4F). Significantly, the defect of pathogenicity in *hcm1*Δ was partially rescued when *SRX1* was overexpressed in *hcm1*Δ (Figure 5A,D). Taken together, these results reveal that Hcm1 orchestrates oxidative stress defense and pathogenicity in *C. neoformans* largely through its direct target *SRX1*.

### *HCM1* plays a conserved and important role in antioxidation in different clinical strains of *C. neoformans* but not in those of *C. deuterogattii*

Next, we asked whether Hcm1 likewise activates the expression of *SRX1* in R265. As shown in Figure 5B, a significantly attenuated expression of *SRX1* was observed in the cells where Hcm1 was absent. This finding indicates that *SRX1* is a conserved target of Hcm1 in both H99 and R265. To test the impact of Srx1 on the oxidative stress response and virulence in R265, *SRX1* was knocked out in this background. We first examined two independent *srx1*Δ mutants for their abilities to cause infections. We found that unlike *srx1*Δ from H99 as reported by Upadhya et al.,^43^ R265‐derived *srx1*Δ mutants were as virulent as their parent strain (Figure 5C), indicative of an obvious difference in the contribution of Srx1 to pathogenicity in different *Cryptococcus* pathogens. Consistently, a similar fungal burden in the lungs and brains was observed in mice infected with R265 wild‐type and the corresponding *srx1*Δ mutant strains (Figure 5E).

**Figure 6:**
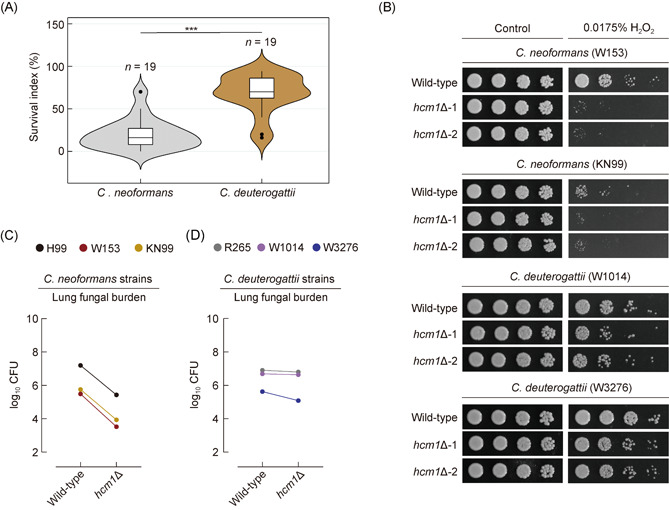
*HCM1* plays a conserved and important role in antioxidation and pathogenicity in different clinical strains of *Cryptococcus neoformans* but not in those of *Cryptococcus deuterogattii*. (A) Semiquantitatively phenotypic assays were performed in the presence of H_2_O_2_ at the indicated concentration. The survival index of *C. neoformans* strains (*n* = 19) and *C. deuterogattii* strains (*n* = 19) were based on data presented in Figure S4. (B) Spotting susceptibility assays of *hcm1*Δ mutants derived from different *C. neoformans* and *C. deuterogattii* strains and their corresponding parent strain were performed in the presence of H_2_O_2_ at the indicated concentration. Data represent the mean ± SD (*n* = 3). (C) Lung fungal burdens from the mice infected with different *C. neoformans* strains and their corresponding *hcm1*Δ mutants at DPI 14. (D) Lung fungal burdens from the mice infected with different *C. deuterogattii* strains and their corresponding *hcm1*Δ mutants at DPI 14. Data represent the mean ± SD (*n* = 3). ***p < 0.001.

We further evaluated the function of Srx1 in cellular tolerance to H_2_O_2_ in R265 and found that although the deletion of *SRX1* also reduced cellular tolerance to H_2_O_2_, the reduction was much lower than that caused by the corresponding mutation in H99 (Figure 5F). Interestingly, our phenotypic assays indicated a much stronger tolerance to H_2_O_2_ of R265 relative to H99 (Figure 5F), and this finding is consistent with the results reported by Ueno et al^44^. Moreover, the resistance of H99 to H_2_O_2_ was relatively similar to that of the R265‐derived *srx1*Δ mutant (Figure 5F). Next, we asked if the observed difference in oxidative stress tolerance is strain‐specific or species‐specific. Thirty‐eight clinically isolated strains of *C. neoformans* (*n* = 19) and *C. deuterogattii* (*n* = 19) were individually tested for their adaptation to H_2_O_2_. In this experiment, *C. deuterogattii* strains generally exhibited better resistance to H_2_O_2_ than *C. neoformans* isolates (Figures 6A and S4A,B). This result supports the notion that *C. deuterogattii* species is more tolerant to oxidative stress than *C. neoformans* species, which may reflect the genetic redundancy of *C. deuterogattii* in antioxidation.

To test this idea, we knocked out *HCM1* in different clinical strains of *C. neoformans* and *C. deuterogattii*. The corresponding mutants were subjected to phenotypic assays for evaluating their growth fitness in the presence of H_2_O_2_ or during infections. Similar to the observation in H99, all *C. neoformans*‐derived *hcm1*Δ mutants showed severe growth defects in the presence of H_2_O_2_ (Figure 6B). By comparison, in the presence of H_2_O_2_ at the same concentration, *C. deuterogattii* strains in which *HCM1* was deleted showed largely unchanged growth (Figure 6B). We further tested the role of Hcm1 in virulence in different *C. neoformans* and *C. deuterogattii* strains. For *C. neoformans* strains, the lung fungal burdens in mice infected with the *hcm1*Δ mutants were significantly lower than those inoculated with their parent strains (Figure 6C). In contrast, mutating *HCM1* in *C. deuterogattii* strains led to modest or undetectable impairment of cryptococcal survival in the lungs (Figure 6D). Altogether, these results show a dramatic difference in the contribution of *HCM1* to antioxidation and pathogenicity in different *Cryptococcus* species.

## DISCUSSION

Despite a close evolutionary relationship, *C. neoformans* and *C. deuterogattii* have disparate clinical characteristics^4^. *C. neoformans* primarily infects immunodeficient populations, such as AIDS patients, and often cause central nervous system infections^4^, ^45^. However, *C. deuterogattii* is frequently associated with infections in immunocompetent populations and can cause severe lung disease without dissemination to other organs^46^, ^47^. These dissimilarities indicate that these pathogens may employ different molecular machines and regulatory systems for enabling their pathogenicity. An earlier study performed by Lam et al. demonstrated that the chitin deacetylase Cda1 is essential for virulence in *C. neoformans* KN99, while in *C. deuterogattii* R265, Cda3 rather than Cda1 is the major chitin deacetylase engaged in pathogenicity^48^. Most recently, Lee et al. showed that the homeobox transcription factor Hob1 facilitates brain infection in H99, but its homolog in R265 does not^49^. Despite these important findings, the determinants and mechanisms underlying the interspecies regulatory differences of pathogenicity remain unclear.

Our data indicated that the regulatory differences are partially attributed to the forkhead transcription factor Hcm1. Due to sharing a modest similarity with the cell cycle regulator Hcm1 from the ascomycete *S. cerevisiae*, this regulator was considered to play a similar role in cell cycle control in the basidiomycete *C. neoformans*. However, results of our phenotypic and transcriptomic analyses showed that Hcm1 does not play role in the regulation of the cell cycle in either *C. neoformans* or *C. deuterogattii*, indicating a disparity of function between Hcm1 orthologs from *S. cerevisiae* and *Cryptococcus* pathogens, which diverged 500 million years ago^50^. We further revealed the importance of Hcm1 in antioxidation in *C. neoformans*. The Hcm1‐directed orchestration of the oxidative stress response and pathogenicity in this pathogen largely depends on its direct regulation of sulfiredoxin‐coding gene *SRX1*, which is dedicated to reducing inorganic and organic peroxides through recycling of peroxiredoxins in various fungi, including *C. neoformans* ^40^, ^41^. Interestingly, although the regulatory relationship between Hcm1 and *SRX1* was also identified in *C. deuterogattii* R265, neither of them is required for virulence in this pathogen. Phenotypic analyses revealed that the contribution of Hcm1 and Srx1 to oxidative stress defense in R265 is obviously weaker than their homologs in H99. Of note, R265 showed a much stronger tolerance to hydrogen peroxide than H99. This finding is consistent with the previous observation that R265 has a better fitness in macrophages than H99 due to its more potent ROS tolerance^26^, ^27^, ^28^. We further revealed that the distinction in ROS defense is not strain‐specific but species‐dependent. By monitoring the fitness of dozens of clinical isolates from two species, it was found that *C. deuterogattii* strains generally are robust in ROS tolerance compared with isolates of *C. neoformans*. This suggests the possibility that the genetic redundancy of antioxidation in *C. deuterogattii* may be related to a species‐specific feature regarding strong ROS tolerance. Supporting this, the wild‐type‐like antioxidant capability was detected upon deletion of *HCM1* in *C. deuterogattii* clinical strains in the presence of high H_2_O_2_, which otherwise resulted in a dramatic reduction in growth fitness in *hcm1*Δ mutants generated from *C. neoformans* strains.

Clinically, *C. deuterogattii* is considered to be more virulent than *C. neoformans* in terms of lung infection^51^, ^52^, ^53^. It has been well recognized that during lung infection, the survival of pathogens is highly associated with their abilities to defend against ROS released by immune cells, such as macrophages^36^, ^38^. In this regard, the better adaptation to ROS by *C. deuterogattii* may partially explain its excellent capability for enabling lung infection. Further dissection of species‐specific gene networks underlying antioxidation in these two *Cryptococcus* species may provide important insights into how their pathogenicity features during lung infections are specified and may indicate novel targets for the development of antifungals against these two important fungal pathogens.

## MATERIALS AND METHODS

### Strains and growth conditions

The strains used in this study are listed in Table S1. *C. neoformans* and *C. deuterogattii* were cultured on yeast extract–peptone–dextrose (YPD) liquid medium (1% yeast extract, 2% Bacto peptone, 2% dextrose, and 2% Bacto agar) at 30°C unless specified otherwise. Dulbecco's modified Eagle's (DME) medium was used to measure capsule production at 37°C in 5% CO_2_ ^54^. Minimal medium (MM) containing L‐dihydroxyphenylalanine (L‐DOPA) was used to test melanin production at 30°C in the dark^55^.

### Gene disruption and overexpression

The primers used in this study are listed in Table S2. Targeted gene deletion was performed as previously described^56^. Briefly, overlapping PCR products were generated with nourseothricin (NAT) or neomycin (NEO) resistance cassettes and 5′ and 3′ flanking sequences (1.0–1.5 kb) of the target genes from *C. neoformans* and *C. deuterogattii* strains as previously described. The PCR products were introduced into relevant recipient strains by the TRACE method^35^. Genotypic accuracy of the positive transformants was confirmed by PCR. For gene overexpression, genes (open reading frame—ORF) were amplified by PCR and were cloned into the plasimd pXC^56^ with a FLAG tag behind the P_*CnH3*_ promoter and introduced into the safe haven regions^57^ of relevant recipient strains by electroporation.

### Growth curve assay

Cells were inoculated into culture tubes containing YPD liquid medium at a starting cell density of *A*_600_ = 0.1 (*A*_600_: absorbance at 600 nm). Cells were incubated at 30°C and 37°C with shaking at the rate of 220 rpm. The cell concentration was measured at the indicated time points with an ultraviolet–visible spectrophotometer.

### Phylogenetic analysis

The Hcm1 protein sequence from *C. neoformans* H99 was used as the query sequence for Basic Local Alignment Search Tool (BLAST) search. The phylogenetic trees for Hcm1 homologs were developed with their whole‐protein sequences by using the neighbor‐joining method in MEGA‐X.

### Flow cytometry analysis

Analysis of the *Cryptococcus* cell cycle by flow cytometry was conducted according to a method described previously^58^, ^59^. Briefly, *Cryptococcus* strains were collected at different time points (i.e., 6, 12, and 24 h) after transferring the overnight cultures to fresh YPD liquid medium, then washed twice in phosphate‐buffered saline (PBS) and fixed in 70% ethanol overnight at 4°C. Fixed cells were washed once with NS buffer (10 mM Tris–HCl [pH 7.6], 250 mM sucrose, 1 mM ethylenediaminetetraacetic acid [pH 8.0], 1 mM MgCl_2_, 0.1 mM CaCl_2_, 0.1 mM ZnCl_2_, 0.4 mM phenylmethylsulfonyl fluoride, and 7 mM β‐mercaptoethanol). Cells were stained with propidium iodide (12.5 μg/ml) in NS buffer containing RNase A (1.0 mg/ml). The mixture was incubated with agitation at room temperature for 2–3 h. Stained cells were diluted into Tris–HCl (pH = 8.0) buffer and sonicated for 1 min. Finally, 10,000 cells were analyzed on the FL2 channel on a BD FACSCalibur at the Beijing Regional Center of Life Science Instrument, Chinese Academy of Sciences.

### Growth and chemical susceptibility analyses

Growth and chemical susceptibility analyses were performed as described previously^11^. Briefly, cells were cultured overnight in YPD liquid medium at 30°C with shaking at 220 rpm, and then washed twice with PBS, adjusted to the same cell density (5.0 × 10^7^ CFU/ml), and were five‐fold serially diluted. To test growth phenotypes of different *Cryptococcus* cells at distinct temperatures, an equal number of cells were spotted onto YPD solid medium and grown at 25, 30, 37°C, 37°C + 5% CO_2_, and 39°C. To analyze abiotic stress‐related phenotypes of different *Cryptococcus* species, the diluted cells were spotted onto YPD solid medium containing the indicated concentrations of the following chemical reagents: 1.5 M NaCl and 1.5 M KCl, 4% Congo red and 0.03% sodium dodecyl sulfate, 1.5 mM *tert*‐butyl hydroperoxide, 5 mM diamide and 50 μM menadione, 0.6 μg/ml amphotericin B, 300 μg/ml 5‐flucytosine, 20 μg/ml fluconazole, 0.1 μg/ml miconazole, 500 ng/ml rapamycin, 2.0 μg/ml fludioxonil, 2 M sorbitol, 0.1 μg/ml tunicamycin, and 0.5 μM CdSO_4_. Cells were incubated at 30°C in the dark for 2–3 days and photographed.

### Assays for capsule and melanin production

Capsule production was measured as described previously^54^. Briefly, cells were cultured overnight in YPD liquid medium at 30°C with shaking at 220 rpm, then washed three times with PBS and diluted to a final concentration of 1.0 × 10^5^/ml in a DME medium. To induce capsule formation, the cells were incubated at 37°C with 5% CO_2_ for 72 h. The capsules were visualized by negative staining with India ink under a light microscope. To analyze melanization, cells were cultured overnight in YPD liquid medium at 30°C. The cells were washed twice with PBS and then adjusted to the same cell density (5.0 × 10^7^ CFU/ml) and fivefold serially diluted. The diluted cells were spotted on MM containing L‐DOPA, incubated at 30°C for 3 days in the dark, and photographed^55^.

### Murine model of cryptococcosis

Mouse lung infections were performed as previously described^8^, ^60^. Briefly, female C57 mice (7–8 weeks old) were subjected to cryptococcal infection by inhalation. *Cryptococcal* strains were grown in YPD liquid medium overnight at 30°C with shaking at 220 rpm. The fungal cells were washed twice with PBS and adjusted to 2.0 × 10^6^ CFU/ml. For survival assays, mice (*n* = 10) were randomly distributed into each group. The mice were anesthetized with ketamine and xylazine and then infected with 50 μl of fungal cell suspensions via intranasal instillation. After infection, animals were observed daily for disease progression, including weight changes and labored breaths.

### Organ fungal burden analysis

For organ fungal burden analysis, the infection procedure was the same as for the survival assay. The lungs and brains of three infected mice at DPI 14 were homogenized using a homogenizer and spread on a YPD solid medium containing 100 μg/ml of chloramphenicol. The plates were incubated at 30°C for 2–3 days, and then CFUs were counted manually.

### Histology assays

Histology assays were performed as described previously^61^, ^62^. Briefly, mice infected with different *Cryptococcus* strains were killed at DPI 14. The lungs were harvested and fixed in 10% formalin solution and sent to the Peking Union Medical College Hospital for section preparation. Tissue slides were stained with hematoxylin and eosin (H&E) staining and examined by light microscopy.

### RNA‐seq and data analysis

For the RNA‐seq analysis, the wild‐type H99 and its corresponding *hcm1*Δ mutant strain were cultured in a YPD liquid medium at 30°C for 6 h. RNA extraction was performed as previously described^56^, ^63^, ^64^. Briefly, total RNA was extracted using Ultrapure RNA Kit (CW0581M, CWBIO) according to the manufacturer's instructions. RNA‐seq and data analysis were performed as previously described. Briefly, RNA‐seq was performed by Annoroad Gene Technology Co., Ltd. Initial quality control of sequenced clean data was performed using FastQC v0.11.5 software. Then, Hisat2 v2.1.0 was used for clean short reads mapping to the genome sequence of *C. neoformans* H99. The relative transcript abundances were measured in transcripts per million by Stingtie v1.3.3 to determine unigenes. DEGs were identified with DEseq. 2 and defined based on the basis of the fold change criterion (|log_2_(fold change)| > 1.0, *q* < 0.05).

### Quantitative RT‐PCR analysis

To test whether *SRX1* is regulated by Hcm1 both in *C. neoformans* H99 and *C. deuterogattii* R265 backgrounds. H99 and R265 wild‐type and corresponding *hcm1*Δ mutants were cultured in YPD liquid medium overnight at 30°C with shaking at 220 rpm and then transferred to RPMI 1640 medium at 37°C with 5% CO_2_ to mimic host physiological environment. The cells were collected at 6 h postincubation for the isolation of total RNA. Total RNA was extracted using an Ultrapure RNA Kit (CW0581M, CWBIO) according to the manufacturer's instructions. The RNA was reverse‐transcribed with the Fastquant RT Kit (with gDNase, KR106‐02, Tiangen). The relative mRNA level of *SRX1* was calculated by real‐time RT‐PCR. The relative transcript levels of *SRX1* were normalized to that of the constitutively expressed housekeeping gene *TEF1* and determined by the comparative Ct method as described previously^54^.

### ChIP‐assay

ChIP‐assays were performed as described previously^65^, ^66^, ^67^. Briefly, cells were cultured in RPMI 1640 medium for 6 h in an incubator containing 5% CO_2_ at 37°C. Cells were then harvested and cross‐linked with 1% formaldehyde. Glycine (final concentration 125 mM) was subsequently added to quench the cross‐linking reaction. Micrococcal nuclease was used to fragment chromatin to an appropriate size. Clarified chromatin extracts were immunoprecipitated with an anti‐FLAG antibody. The DNA–protein complex was then washed and eluted, and eluted samples were subjected to reversal of cross‐linking. Afterward, DNA was purified by the phenol–chloroform extraction method and precipitated in ethanol. Primers upstream of the *SRX1* ORF were used to perform qPCR. The ChIP enrichment signal was quantified as the percentage of input. Primers used in ChIP‐qPCR analyses are listed in Table S2.

### Statistical analysis

Statistical analyses were performed with Prism 8.0. We used two‐tailed Student's *t* tests to compare lung and brain fungal burden, cell size, survival index, or transcript levels from two groups. Gehan–Breslow–Wilcoxon tests were utilized to evaluate the significance of survival data for the various groups. For all analyses, *p* value less than 0.05 (typically <0.05) is statistically significant and *p* value higher than 0.05 (>0.05) is not statistically significant. In all figures, the data are shown as mean ± standard deviation (SD) from three or more independent biological replicates.

## AUTHOR CONTRIBUTIONS

Weixin Ke conducted and designed most of the studies and wrote the original draft; Yuyan Xie conducted part of the gene deletion and comparative phenotypic analysis experiments and wrote the original draft; Yue Hu conducted the chromatin immunoprecipitation (ChIP) assay; Hao Ding conducted most of the bioinformatics assays; Xiuyun Tian conducted the RT‐PCR; Xin Fan conducted part of the comparative phenotypic analysis; Jingjing Huang conducted histology assays; Yingchun Xu, Xiao Liu, and Ying Yang contributed reagents, materials, and resources. Linqi Wang wrote the original draft and was responsible for project administration, writing—review and editing.

## ETHICS STATEMENT

All experiments involving mice were performed under the guidance of “the regulation of the Institute of Microbiology, Chinese Academy of Sciences of Research Ethics Committee.” The mouse models and procedures performed have been approved by the Institute of Microbiology, Chinese Academy of Sciences of Research Ethics Committee (Permit No. APIMCAS2021146).

## CONFLICT OF INTERESTS

The authors declare that they have no conflict of interests to this work.

## Supporting information

Supporting information.

Supporting information.

Supporting information.

Supporting information.

## Data Availability

The data that support the findings of this study are available from the corresponding author upon request.

## References

[1] Zhao RL , Li GJ , Sánchez‐Ramírez S , Stata M , Yang ZL , Wu G , et al. A six‐gene phylogenetic overview of Basidiomycota and allied phyla with estimated divergence times of higher taxa and a phyloproteomics perspective. Fungal Diversity. 2017;84:43–74.

[2] Kirk P , Cannon P , David J , Stalpers J . Dictionary of the fungi. 10th ed. Wallingford: CABI; 2008.

[3] Kwon‐Chung K , Boekhout T , Fell J , Diaz M . (1557) Proposal to conserve the name *Cryptococcus gattii* against *C. hondurianus* and *C. bacillisporus* (Basidiomycota, Hymenomycetes, Tremellomycetidae). Taxon. 2002;51:804–6.

[4] Kwon‐Chung KJ , Fraser JA , Doering TL , Wang Z , Janbon G , Idnurm A , et al. *Cryptococcus neoformans* and *Cryptococcus gattii*, the etiologic agents of cryptococcosis. Cold Spring Harb Perspect Med. 2014;4:a019760. 24985132 10.1101/cshperspect.a019760 PMC4066639

[5] Cox GM , Mukherjee J , Cole GT , Casadevall A , Perfect JR . Urease as a virulence factor in experimental cryptococcosis. Infect Immun. 2000;68:443–8. 10639402 10.1128/iai.68.2.443-448.2000 PMC97161

[6] Esher SK , Zaragoza O , Alspaugh JA . Cryptococcal pathogenic mechanisms: a dangerous trip from the environment to the brain. Mem Inst Oswaldo Cruz. 2018;113:e180057. 29668825 10.1590/0074-02760180057 PMC5909089

[7] Oliveira Fde M , Severo CB , Guazzelli LS , Severo LC . *Cryptococcus gattii* fungemia: report of a case with lung and brain lesions mimicking radiological features of malignancy. Rev Inst Med Trop Sao Paulo. 2007;49:263–5. 17823759 10.1590/s0036-46652007000400014

[8] Chun CD , Madhani HD . Applying genetics and molecular biology to the study of the human pathogen *Cryptococcus neoformans* . Methods Enzymol. 2010;470:797–831. 20946836 10.1016/S0076-6879(10)70033-1 PMC3611884

[9] Goranov AI , Madhani HD . Functional profiling of human fungal pathogen genomes. Cold Spring Harb Perspect Med. 2014;5:a019596. 25377143 10.1101/cshperspect.a019596 PMC4355250

[10] Liu OW , Chun CD , Chow ED , Chen C , Madhani HD , Noble SM . Systematic genetic analysis of virulence in the human fungal pathogen *Cryptococcus neoformans* . Cell. 2008;135:174–88. 18854164 10.1016/j.cell.2008.07.046 PMC2628477

[11] Jung KW , Yang DH , Maeng S , Lee KT , So YS , Hong J , et al. Systematic functional profiling of transcription factor networks in *Cryptococcus neoformans* . Nat Commun. 2015;6:6757. 25849373 10.1038/ncomms7757 PMC4391232

[12] Lee KT , So YS , Yang DH , Jung KW , Choi J , Lee DG , et al. Systematic functional analysis of kinases in the fungal pathogen *Cryptococcus neoformans* . Nat Commun. 2016;7:12766. 27677328 10.1038/ncomms12766 PMC5052723

[13] Zhao Y , Lin J , Fan Y , Lin X . Life cycle of *Cryptococcus neoformans* . Annu Rev Microbiol. 2019;73:17–42. 31082304 10.1146/annurev-micro-020518-120210 PMC12860491

[14] Lin X , Heitman J . The biology of the *Cryptococcus neoformans* species complex. Annu Rev Microbiol. 2006;60:69–105. 16704346 10.1146/annurev.micro.60.080805.142102

[15] Petter R , Kang BS , Boekhout T , Davis BJ , Kwon‐Chung KJ . A survey of heterobasidiomycetous yeasts for the presence of the genes homologous to virulence factors of *Filobasidiella neoformans*, CNLAC1 and CAP59. Microbiology. 2001;147:2029–36. 11495981 10.1099/00221287-147-8-2029

[16] Perfect JR . *Cryptococcus neoformans*: the yeast that likes it hot. FEMS Yeast Res. 2006;6:463–8. 16696642 10.1111/j.1567-1364.2006.00051.x

[17] Chang YC , Kwon‐Chung KJ . Complementation of a capsule‐deficient mutation of *Cryptococcus neoformans* restores its virulence. Mol Cell Biol. 1994;14:4912–9. 8007987 10.1128/mcb.14.7.4912-4919.1994 PMC358863

[18] Kwon‐Chung KJ , Polacheck I , Popkin TJ . Melanin‐lacking mutants of *Cryptococcus neoformans* and their virulence for mice. J Bacteriol. 1982;150:1414–21. 6804444 10.1128/jb.150.3.1414-1421.1982 PMC216368

[19] Farrer RA , Voelz K , Henk DA , Johnston SA , Fisher MC , May RC , et al. Microevolutionary traits and comparative population genomics of the emerging pathogenic fungus *Cryptococcus gattii* . Philos Trans R Soc Lond B Biol Sci. 2016;371:20160021. 28080992 10.1098/rstb.2016.0021 PMC5095545

[20] Ngamskulrungroj P , Gilgado F , Faganello J , Litvintseva AP , Leal AL , Tsui KM , et al. Genetic diversity of the *Cryptococcus* species complex suggests that *Cryptococcus gattii* deserves to have varieties. PLoS One. 2009;4:e5862. 19517012 10.1371/journal.pone.0005862 PMC2690690

[21] Chen Y , Farrer RA , Giamberardino C , Sakthikumar S , Jones A , Yang T , et al. Microevolution of serial clinical isolates of *Cryptococcus neoformans* var. *grubii* and *C. gattii* . mBio. 2017;8:e00166‐17. 28270580 10.1128/mBio.00166-17 PMC5340869

[22] Montoya MC , Magwene PM , Perfect JR . Associations between *Cryptococcus* genotypes, phenotypes, and clinical parameters of human disease: a review. J Fungi (Basel). 2021;7:260. 33808500 10.3390/jof7040260 PMC8067209

[23] van Spil WE , Nooijen S , de Jong PY , Aliredjo RP , de Sevaux RG , Verhave JC . Cryptococcal meningitis. Ned Tijdschr Geneeskd. 2015;159:A8478. 25827149

[24] McMullan BJ , Sorrell TC , Chen SC . *Cryptococcus gattii* infections: contemporary aspects of epidemiology, clinical manifestations and management of infection. Future Microbiol. 2013;8:1613–31. 24266360 10.2217/fmb.13.123

[25] Schotanus K , Heitman J . Centromere deletion in *Cryptococcus deuterogattii* leads to neocentromere formation and chromosome fusions. eLife. 2020;9:e56026. 32310085 10.7554/eLife.56026 PMC7188483

[26] Voelz K , Johnston SA , Smith LM , Hall RA , Idnurm A , May RC . 'Division of labour' in response to host oxidative burst drives a fatal *Cryptococcus gattii* outbreak. Nat Commun. 2014;5:5194. 25323068 10.1038/ncomms6194 PMC4208095

[27] Gibson JF , Johnston SA . Immunity to *Cryptococcus neoformans* and *C. gattii* during cryptococcosis. Fungal Genet Biol. 2015;78:76–86. 25498576 10.1016/j.fgb.2014.11.006 PMC4503824

[28] Ma H , Hagen F , Stekel DJ , Johnston SA , Sionov E , Falk R , et al. The fatal fungal outbreak on Vancouver Island is characterized by enhanced intracellular parasitism driven by mitochondrial regulation. Proc Natl Acad Sci USA. 2009;106:12980–5. 19651610 10.1073/pnas.0902963106 PMC2722359

[29] Ghavidel A , Baxi K , Prusinkiewicz M , Swan C , Belak ZR , Eskiw CH , et al. Rapid nuclear exclusion of Hcm1 in aging *Saccharomyces cerevisiae* leads to vacuolar alkalization and replicative senescence. G3 (Bethesda). 2018;8:1579–92. 29519938 10.1534/g3.118.200161 PMC5940150

[30] Negishi T , Veis J , Hollenstein D , Sekiya M , Ammerer G , Ohya Y . The late S‐phase transcription factor Hcm1 Is regulated through phosphorylation by the cell wall integrity checkpoint. Mol Cell Biol. 2016;36:941–53. 26729465 10.1128/MCB.00952-15 PMC4810473

[31] Pramila T , Wu W , Miles S , Noble WS , Breeden LL . The forkhead transcription factor Hcm1 regulates chromosome segregation genes and fills the S‐phase gap in the transcriptional circuitry of the cell cycle. Genes Dev. 2006;20:2266–78. 16912276 10.1101/gad.1450606 PMC1553209

[32] Arsenault HE , Roy J , Mapa CE , Cyert MS , Benanti JA . Hcm1 integrates signals from Cdk1 and calcineurin to control cell proliferation. Mol Biol Cell. 2015;26:3570–7. 26269584 10.1091/mbc.E15-07-0469 PMC4603928

[33] Rodriguez Colman MJ , Ros J , Cabiscol E . Mitochondrial localization of the yeast forkhead factor Hcm1. Int J Mol Sci. 2020;21:9574. 33339134 10.3390/ijms21249574 PMC7765673

[34] Benanti JA . Create, activate, destroy, repeat: Cdk1 controls proliferation by limiting transcription factor activity. Curr Genet. 2016;62:271–6. 26590602 10.1007/s00294-015-0535-5

[35] Fan Y , Lin X . Multiple applications of a transient CRISPR‐Cas9 coupled with electroporation (TRACE) system in the *Cryptococcus neoformans* species complex. Genetics. 2018;208:1357–72. 29444806 10.1534/genetics.117.300656 PMC5887135

[36] Upadhya R , Campbell LT , Donlin MJ , Aurora R , Lodge JK . Global transcriptome profile of *Cryptococcus neoformans* during exposure to hydrogen peroxide induced oxidative stress. PLoS One. 2013;8:e55110. 23383070 10.1371/journal.pone.0055110 PMC3557267

[37] Missall TA , Pusateri ME , Lodge JK . Thiol peroxidase is critical for virulence and resistance to nitric oxide and peroxide in the fungal pathogen, *Cryptococcus neoformans* . Mol Microbiol. 2004;51:1447–58. 14982637 10.1111/j.1365-2958.2004.03921.x

[38] de Jesus‐Berrios M , Liu L , Nussbaum JC , Cox GM , Stamler JS , Heitman J . Enzymes that counteract nitrosative stress promote fungal virulence. Curr Biol. 2003;13:1963–8. 14614821 10.1016/j.cub.2003.10.029

[39] Cheon SA , Thak EJ , Bahn YS , Kang HA . A novel bZIP protein, Gsb1, is required for oxidative stress response, mating, and virulence in the human pathogen *Cryptococcus neoformans* . Sci Rep. 2017;7:4044. 28642475 10.1038/s41598-017-04290-8 PMC5481450

[40] Roussel X , Kriznik A , Richard C , Rahuel‐Clermont S , Branlant G . Catalytic mechanism of sulfiredoxin from *Saccharomyces cerevisiae* passes through an oxidized disulfide sulfiredoxin intermediate that is reduced by thioredoxin. J Biol Chem. 2009;284:33048–55. 19801666 10.1074/jbc.M109.035352 PMC2785145

[41] Gutiérrez‐Escobedo G , Hernández‐Carreón O , Morales‐Rojano B , Revuelta‐Rodríguez B , Vázquez‐Franco N , Castaño I , et al. *Candida glabrata* peroxiredoxins, Tsa1 and Tsa2, and sulfiredoxin, Srx1, protect against oxidative damage and are necessary for virulence. Fungal Genet Biol. 2020;135:103287. 31654781 10.1016/j.fgb.2019.103287

[42] Vivancos AP , Jara M , Zuin A , Sanso M , Hidalgo E . Oxidative stress in *Schizosaccharomyces pombe*: different H_2_O_2_ levels, different response pathways. Mol Genet Genomics. 2006;276:495–502. 17043891 10.1007/s00438-006-0175-z

[43] Upadhya R , Kim H , Jung KW , Park G , Lam W , Lodge JK , et al. Sulphiredoxin plays peroxiredoxin‐dependent and ‐independent roles via the HOG signalling pathway in *Cryptococcus neoformans* and contributes to fungal virulence. Mol Microbiol. 2013;90:630–48. 23998805 10.1111/mmi.12388 PMC3943550

[44] Ueno K , Yanagihara N , Otani Y , Shimizu K , Kinjo Y , Miyazaki Y . Neutrophil‐mediated antifungal activity against highly virulent *Cryptococcus gattii* strain R265. Med Mycol. 2019;57:1046–54. 30668754 10.1093/mmy/myy153

[45] Fang W , Fa Z , Liao W . Epidemiology of *Cryptococcus* and cryptococcosis in China. Fungal Genet Biol. 2015;78:7–15. 25445309 10.1016/j.fgb.2014.10.017

[46] Saijo T , Chen J , Chen SC , Rosen LB , Yi J , Sorrell TC , et al. Anti‐granulocyte‐macrophage colony‐stimulating factor autoantibodies are a risk factor for central nervous system infection by *Cryptococcus gattii* in otherwise immunocompetent patients. mBio. 2014;5:e00912‐14. 24643864 10.1128/mBio.00912-14 PMC3967522

[47] Sorrell TC , Chen SC‐A , Phillips P , Marr KA . Clinical perspectives on *Cryptococcus neoformans* and *Cryptococcus gattii*: implications for diagnosis and management. In: Heitman J , Kozel TR , Kwon‐Chung KJ , Perfect JR , Casadevall A , editors. Cryptococcus. 1st ed. Washington, DC: ASM Press; 2010. p. 595–606.

[48] Lam WC , Upadhya R , Specht CA , Ragsdale AE , Hole CR , Levitz SM , et al. Chitosan biosynthesis and virulence in the human fungal pathogen *Cryptococcus gattii* . mSphere. 2019;4:e00644‐194. 10.1128/mSphere.00644-19 PMC6796976 31597720

[49] Lee KT , Hong J , Lee DG , Lee M , Cha S , Lim YG , et al. Fungal kinases and transcription factors regulating brain infection in *Cryptococcus neoformans* . Nat Commun. 2020;11:1521. 32251295 10.1038/s41467-020-15329-2 PMC7090016

[50] Taylor TN , Hass H , Kerp H . The oldest fossil ascomycetes. Nature. 1999;399:648. 10385115 10.1038/21349

[51] Mitchell DH , Sorrell TC , Allworth AM , Heath CH , McGregor AR , Papanaoum K , et al. Cryptococcal disease of the CNS in immunocompetent hosts: influence of cryptococcal variety on clinical manifestations and outcome. Clin Infect Dis. 1995;20:611–6. 7756484 10.1093/clinids/20.3.611

[52] Chen S , Sorrell T , Nimmo G , Speed B , Currie B , Ellis D , et al. Epidemiology and host‐ and variety‐dependent characteristics of infection due to *Cryptococcus neoformans* in Australia and New Zealand. Australasian Cryptococcal Study Group. Clin Infect Dis. 2000;31:499–508. 10987712 10.1086/313992

[53] Galanis E , Macdougall L , Kidd S , Morshed M . British Columbia *Cryptococcus gattii* Working G. Epidemiology of *Cryptococcus gattii*, British Columbia, Canada, 1999–2007. Emerg Infect Dis. 2010;16:251–7. 20113555 10.3201/eid1602.090900 PMC2958008

[54] Liu TB , Wang Y , Stukes S , Chen Q , Casadevall A , Xue C . The F‐Box protein Fbp1 regulates sexual reproduction and virulence in *Cryptococcus neoformans* . Eukaryot Cell. 2011;10:791–802. 21478432 10.1128/EC.00004-11 PMC3127668

[55] Liu TB , Wang Y , Baker GM , Fahmy H , Jiang L , Xue C . The glucose sensor‐like protein Hxs1 is a high‐affinity glucose transporter and required for virulence in *Cryptococcus neoformans* . PLoS One. 2013;8:e64239. 23691177 10.1371/journal.pone.0064239 PMC3653957

[56] Wang L , Zhai B , Lin X . The link between morphotype transition and virulence in *Cryptococcus neoformans* . PLoS Pathog. 2012;8:e1002765. 22737071 10.1371/journal.ppat.1002765 PMC3380952

[57] Upadhya R , Lam WC , Maybruck BT , Donlin MJ , Chang AL , Kayode S , et al. A fluorogenic *C. neoformans* reporter strain with a robust expression of m‐cherry expressed from a safe haven site in the genome. Fungal Genet Biol. 2017;108:13–25. 28870457 10.1016/j.fgb.2017.08.008 PMC5681388

[58] Hu P , Liu L , Ke W , Tian X , Wang L . A cyclin protein governs the infectious and sexual life cycles of *Cryptococcus neoformans* . Sci China Life Sci. 2021;64:1336–45. 33165808 10.1007/s11427-020-1697-3

[59] Tanaka R , Taguchi H , Takeo K , Miyaji M , Nishimura K . Determination of ploidy in *Cryptococcus neoformans* by flow cytometry. J Med Vet Mycol. 1996;34:299–301. 8912162

[60] Lin X , Nielsen K , Patel S , Heitman J . Impact of mating type, serotype, and ploidy on the virulence of *Cryptococcus neoformans* . Infect Immun. 2008;76:2923–38. 18426889 10.1128/IAI.00168-08 PMC2446738

[61] Zhai B , Wozniak KL , Masso‐Silva J , Upadhyay S , Hole C , Rivera A , et al. Development of protective inflammation and cell‐mediated immunity against *Cryptococcus neoformans* after exposure to hyphal mutants. mBio. 2015;6:e01433‐15. 26443458 10.1128/mBio.01433-15 PMC4611043

[62] Sun TS , Ju X , Gao HL , Wang T , Thiele DJ , Li JY , et al. Reciprocal functions of *Cryptococcus neoformans* copper homeostasis machinery during pulmonary infection and meningoencephalitis. Nat Commun. 2014;5:5550. 25417972 10.1038/ncomms6550

[63] Liu L , He GJ , Chen L , Zheng J , Chen Y , Shen L , et al. Genetic basis for coordination of meiosis and sexual structure maturation in *Cryptococcus neoformans* . eLife. 2018;7:e38683. 30281018 10.7554/eLife.38683 PMC6235564

[64] Hu P , Ding H , Shen L , He GJ , Liu H , Tian X , et al. A unique cell wall synthetic response evoked by glucosamine determines pathogenicity‐associated fungal cellular differentiation. PLoS Genet. 2021;17:e1009817. 34624015 10.1371/journal.pgen.1009817 PMC8500725

[65] Tian X , He GJ , Hu P , Chen L , Tao C , Cui YL , et al. *Cryptococcus neoformans* sexual reproduction is controlled by a quorum sensing peptide. Nat Microbiol. 2018;3:698–707. 29784977 10.1038/s41564-018-0160-4 PMC12813696

[66] Homer CM , Summers DK , Goranov AI , Clarke SC , Wiesner DL , Diedrich JK , et al. Intracellular action of a secreted peptide required for fungal virulence. Cell Host Microbe. 2016;19:849–64. 27212659 10.1016/j.chom.2016.05.001 PMC5186401

[67] Skene PJ , Henikoff S . A simple method for generating high‐resolution maps of genome‐wide protein binding. eLife. 2015;4:e09225. 26079792 10.7554/eLife.09225 PMC4480131

