# Supplementary material for: A forkhead transcription factor contributes to the regulatory differences of pathogenicity in closely related fungal pathogens
Source: mLife. 2022 Mar 24;1(1):79–91. doi: 10.1002/mlf2.12011 (PMC10989923; doi:10.1002/mlf2.12011)

**The supporting information files include Figs. S1-S4 and Tables S1-S3.**

**Figure S1. Hcm1 promotes lung damage during infection with *C. neoformans* H99.** (A) Lungs from mice infected with H99 wild-type strain and hcm1Δ mutant strains at DPI 14 were isolated and photographed for phenotypic observation. (B) Hematoxylin and eosin-stained slides were prepared from lung cross-sections infected with H99 wild-type and hcm1Δ mutant strains at DPI 14 and visualized by light microscopy.

**Figure S2. Phenotypes affected by Hcm1 in *C. neoformans* H99 and *C. deuterogattii* R265.** (A) Wild-type (H99) and *hcm1*Δ (H99 background) mutant strains were cultured overnight in liquid YPD medium at 30°C, then 5-fold serially diluted and spotted onto different abiotic stress conditions. Osmotic stress (1.5 M NaCl and 1.5 M KCl), cell wall stress (4% Congo red and 0.03% sodium dodecyl sulfate), oxidative stress (1.5 mM tert-butyl hydroperoxide, 5 mM diamide and 50 μM menadione), antifungal stress (0.6 μg/ml Amphotericin B, 300 μg/ml 5-flucytosine, 20 μg/ml fluconazole, 0.1 μg/ml miconazole, 500 ng/ml rapamycin, 2.0 μg/ml fludioxonil), temperature growth (25°C，30°C，37°C and 39°C), and other stress (2 M sorbitol, 0.1 μg/ml tunicamycin, 0.5 μM CdSO_4_ and 5% CO_2_). (B) Wild-type (R265) and *hcm1*Δ (R265) mutant strains were performed as wild-type (H99) and *hcm1*Δ (H99) mutant strains in the same stress conditions.

**Figure S3. *C. neoformans* Hcm1 affects virulence independently of classical virulence factors.** (A) Melanin production of wild-type and *hcm1*Δ mutants in *C. neoformans* H99 and *C. deuterogattii* R265 backgrounds was performed on minimal medium with or without L-DOPA (100 mg L^–1^) and observed in photographs after incubation in dark for 3 days at 30°C. (B) Growth at 37°C of the wild-type and *hcm1*Δ mutants in *C. neoformans* H99 and *C. deuterogattii* R265 background. (C) Capsule formation was assayed on DME medium at 37°C in 5% CO_2_. Capsule assessment was visualized by India ink staining after cells were cultured on DME medium for 3 days. Scale bar = 5 μm.

**Figure S4. *C. deuterogattii* clinical strains are more tolerant to hydrogen peroxide than those from *C. neoformans*.** (A and B) Spotting susceptibility assays of different *C. neoformans* (A) and *C. deuterogattii* (B) strains were performed in the presence of H_2_O_2_ at the indicated concentration.

**Table S1: Strains used in this study.**

**Table S2: Primers used in this study.**

**Table S3: RNA-seq dataset for wild-type and *hcm1*Δ strains in *C. neoformans* H99 background.**

**Supplementary Figures**

**Figure S1. Hcm1 promotes lung damage during infection with *C. neoformans* H99.**


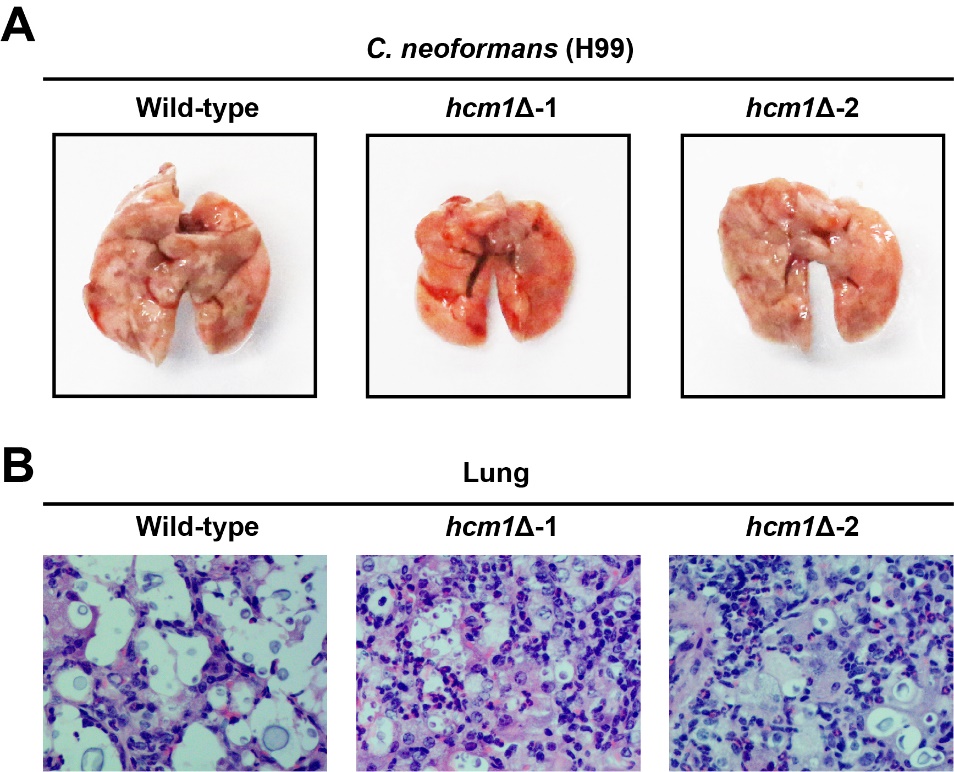


**Figure S2. Phenotypes affected by Hcm1 in *C. neoformans* H99 and *C. deuterogattii* R265.**


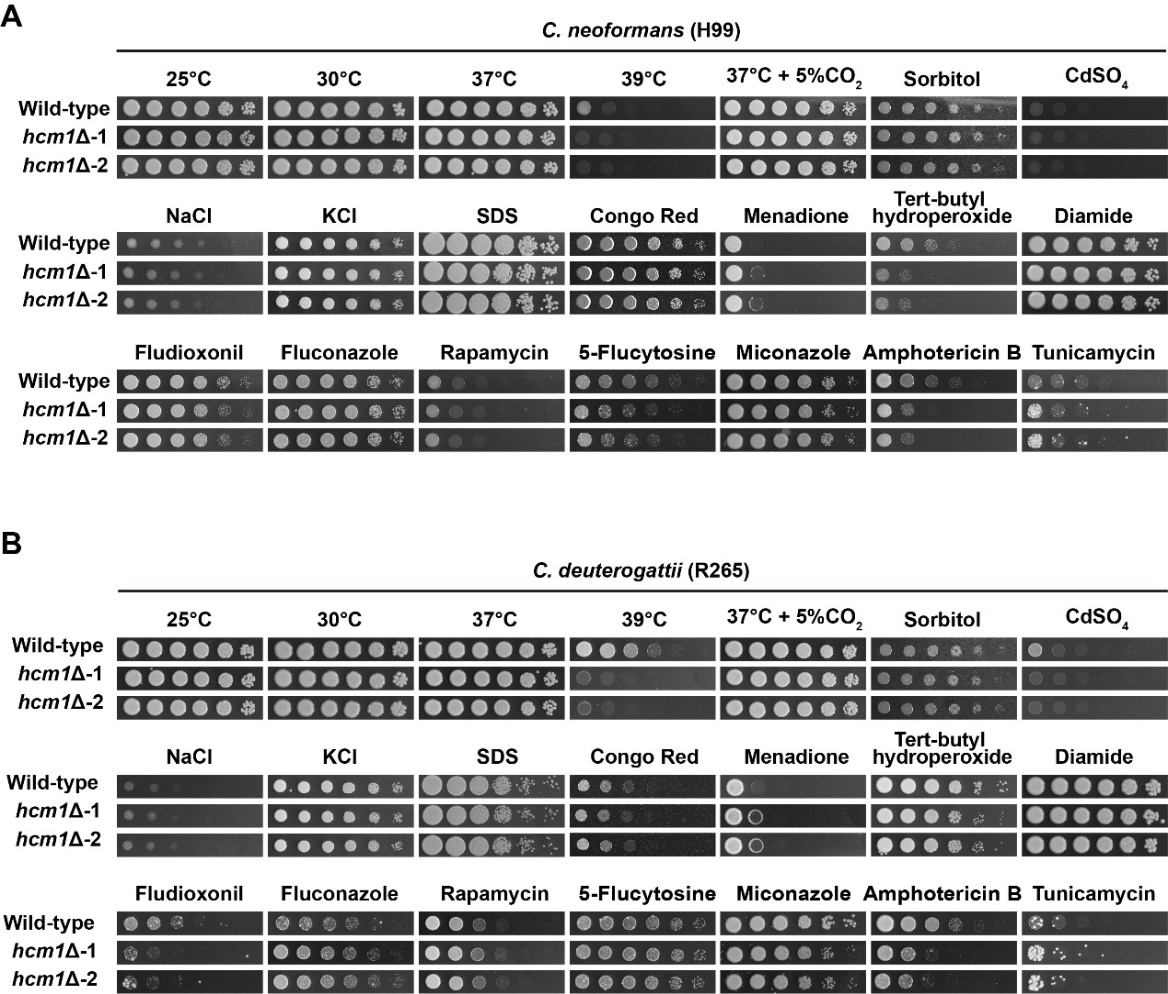


**Figure S3. *C. neoformans* Hcm1 affects virulence independently of classical virulence factors.**


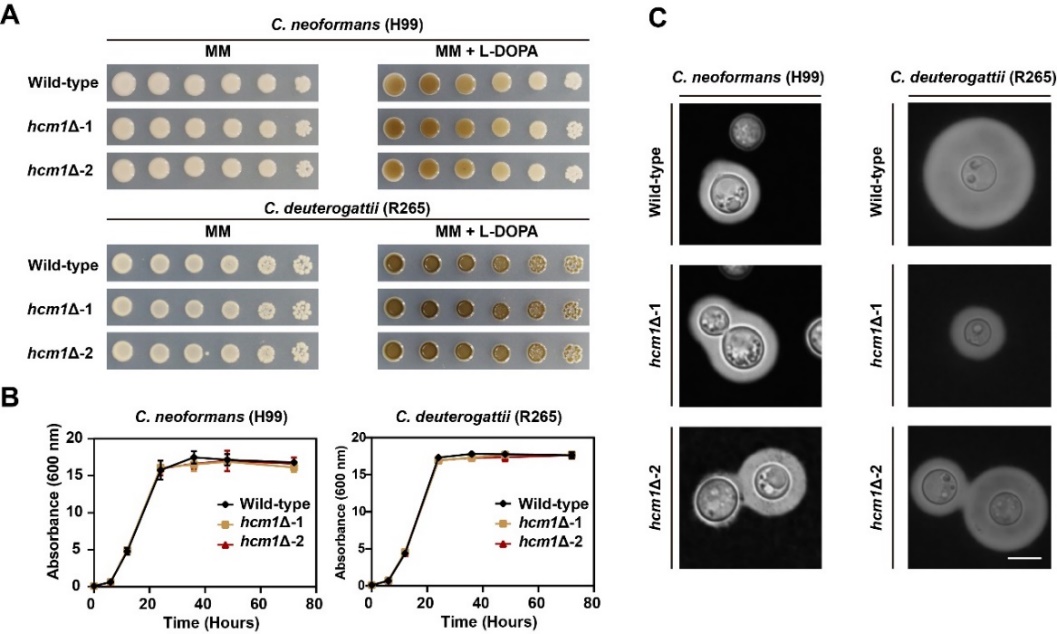


**Figure S4. *C. deuterogattii* clinical strains are more tolerant to hydrogen peroxide than those from *C. neoformans*.**


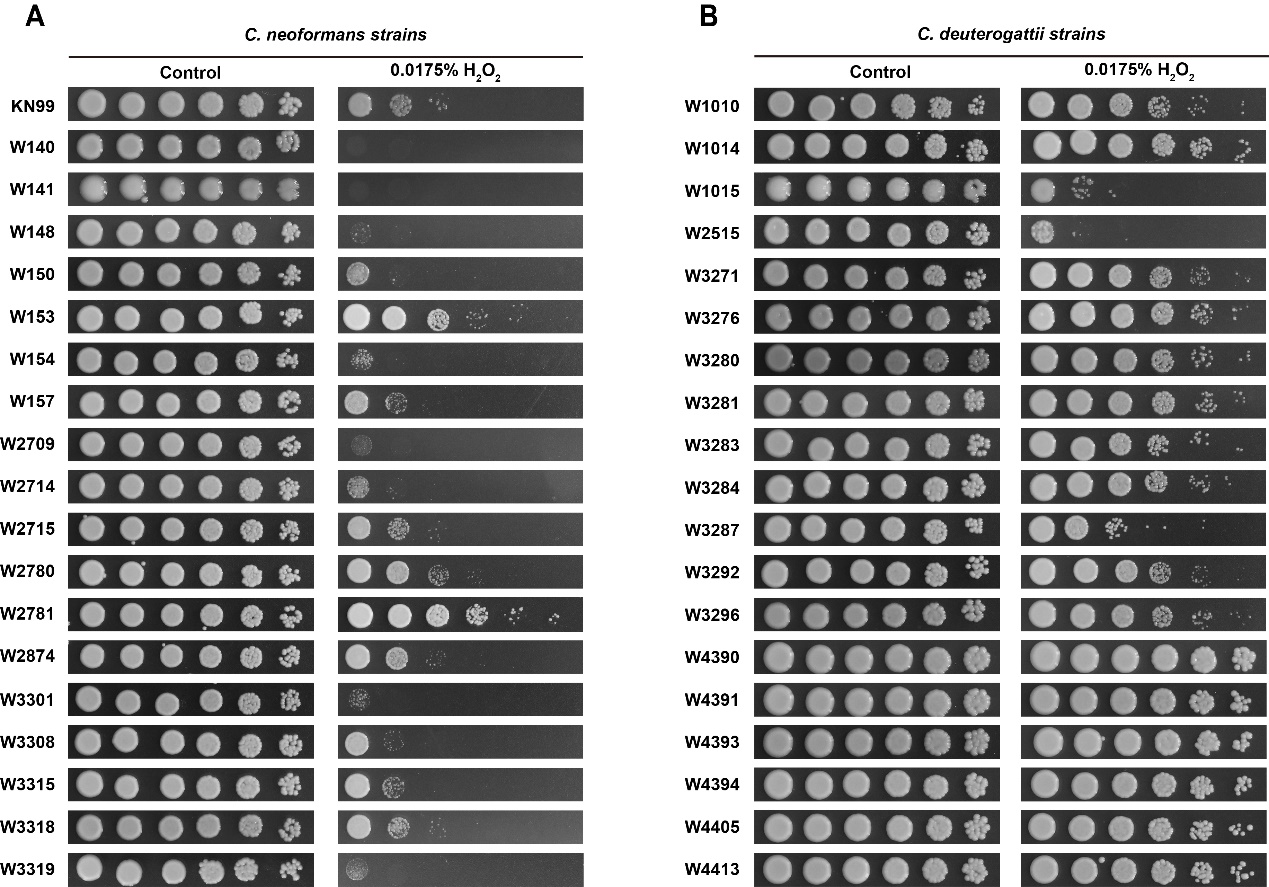

Supplement: Supplementary file 1 — Supporting information. [file MLF2-1-79-s003.docx]
